# Twenty children with non-Wilms renal tumors from a reference center in Central Anatolia, Turkey

**DOI:** 10.3906/sag-1902-106

**Published:** 2020-02-13

**Authors:** Ekrem ÜNAL*, Ebru YILMAZ, Alper ÖZCAN, Bilgen IŞIK, Musa KARAKÜKCÜ, Cuneyt TURAN, Hülya AKGÜN, Figen ÖZTÜRK, Abdulhakim COŞKUN, Mehmet Akif ÖZDEMIR, Türkan PATIROĞLU

**Affiliations:** 1 Division of Pediatric Hematology and Oncology, Department of Pediatrics, Faculty of Medicine, Erciyes University, Kayseri Turkey; 2 Molecular Biology and Genetic Department, Gevher Nesibe Genom and Stem Cell Institution,Genome and Stem Cell Center (GENKÖK), Erciyes University, Kayseri Turkey; 3 Department of Pediatric Surgery, Faculty of Medicine, Erciyes University, Kayseri Turkey; 4 Department of Pathology, Faculty of Medicine, Erciyes University, Kayseri Turkey; 5 Division of Pediatrics Radiology, Department of Radiology, Faculty of Medicine, Erciyes University, Kayseri Turkey

**Keywords:** Non-Wilms renal tumor, children, management

## Abstract

**Background/aim:**

Non-Wilms renal tumors (NWRTs) are rarely encountered in children. The aim of this study is to determine the treatment strategies, prognosis, outcomes, and survival of children with NWRTs at Erciyes University in Kayseri, Turkey.

**Materials and methods:**

Medical records of all patients (n = 20) treated for NWRTs over a 23-year period (1995–2018) were reviewed retrospectively.

**Results:**

There was male predominance (female/male: 7/13); the median age at diagnosis was 3.2 years old (0.1–13.5 years old). The major histological groups included mesoblastic nephroma (MBN), (n: 5, 25%), malignant rhabdoid tumor (MRT), (n: 5, 25%), renal cell carcinoma, (n: 3, 15%), inflammatory myofibroblastic tumor (n: 2, 10%), multilocular cystic renal tumors (n: 2, 10%), metanephric adenoma (n: 1, 5%), renal neuroblastoma (n: 1, 5%), and bilateral renal Ewing sarcoma/primitive neuroectodermal tumor (ES/PNET) (n: 1, 5%). All of the patients with NWRTs had radical nephrectomy except the child with bilateral renal ES/PNET. Six children died because of progressive disease; the mortality rate was 30% (n: 6).

**Conclusion:**

We have made the first report of bilateral renal involvement of ES/PNET in the English medical literature. Physicians dealing with pediatric renal masses should be alert to the high mortality rate in children with MRT, MBN, and ES/PNET and they should design substantial management plans for NWRTs.

## 1. Introduction

Wilms tumor is the most common pediatric renal tumor and accounts for approximately 6%–7% of all pediatric malignancies [1]. Pediatric non-Wilms renal tumors (NWRTs) constitute less than 10% of all renal tumors and have significantly higher mortality rates compared to childhood Wilms tumors [2–8]. There are controversies about the optimal follow-up and treatment plans for this rare and heterogeneous group of tumors. In particular, while the majority of cystic tumors have good prognosis, clinicians should be aware of aggressive tumors such as malignant rhabdoid tumors. In general, NWRTs include mesoblastic nephroma, malignant rhabdoid tumor, renal cell carcinoma, inflammatory myofibroblastic tumor, multilocular cystic renal tumors, and metanephric adenoma. In this work, the experience of pediatric NWRTs at Erciyes University is presented. In addition to the classical tumor subtypes of NWRTs, two interesting cases of renal Ewing sarcoma/primitive neuroectodermal tumor (ES/PNET) and neuroblastoma are also added to this cohort. 

## 2. Materials and methods

This study was carried out in the Department of Pediatric Oncology of Erciyes University in Kayseri, Turkey. Erciyes University Children’s Hospital is a tertiary hospital in the city of Kayseri, in Central Anatolia, Turkey. This hospital is the area’s sole pediatric referral center, serving a wide population, including patients coming from the surrounding cities in the Cappadocia region. Over 23 years (1995–2018), 107 children were managed for renal tumors. The records of 20 (18.7%) non-Wilms’ renal tumors were reviewed. Using patient’s charts, the patient demographics, presenting clinical symptoms and signs, tumor histology, treatment modalities, and outcomes of treatment were analyzed. Ethical permission for a review of all records was granted by the Ethics Committee of Erciyes University (number: 2018-146; date: 21.03.2018).

## 3. Result

**Figure 1:**
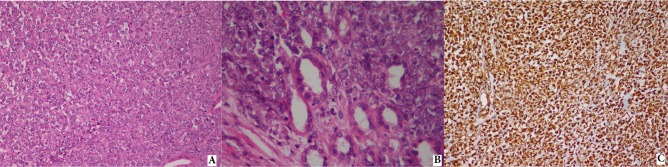
Rhabdoid tumor of the kidney: A) diffuse growth of neoplastic cells (hematoxylin and eosin, 200×), B) sheet-like diffuse pattern of monomorphic neoplastic cells overrunning tubules (hematoxylin and eosin, 400×), C) neoplastic cells are vimentin-positive (vimentin, 200×).

**Figure 2:**
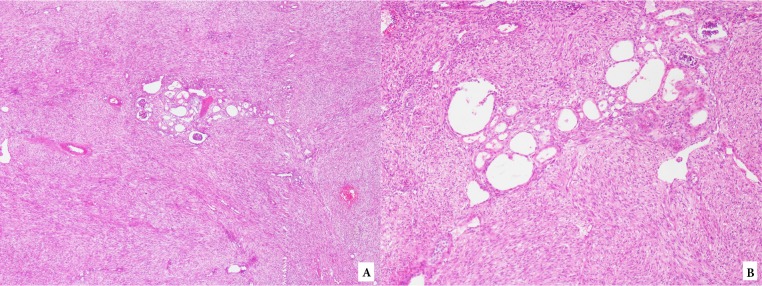
Mesoblastic nephroma: A) fascicles of fibroblastic cells dissect islands of native nephrons (hematoxylin and eosin, 40×), B) fascicles of fibroblastic cells resembling fibromatosis dissect the native kidney (hematoxylin and eosin, 200×).

**Figure 3:**
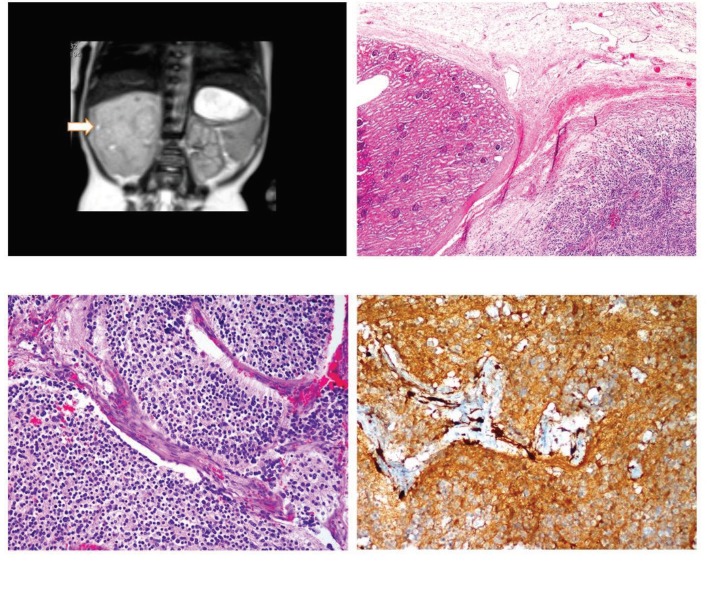
Neuroblastoma of the kidney: A) coronal T2 HASTE image through abdomen demonstrates lobulated hyperintense mass arising from right kidney and infiltrating right adrenal region (arrow), B) neuroblastoma cells adjust to the normal structures of kidney (hematoxylin and eosin, 40×), C) neuroblastoma cells with neurofibrillary background (hematoxylin and eosin, 200×), D) small round cells positive for neuron-specific enolase (neuron-specific enolase, 20×).

**Figure 4:**
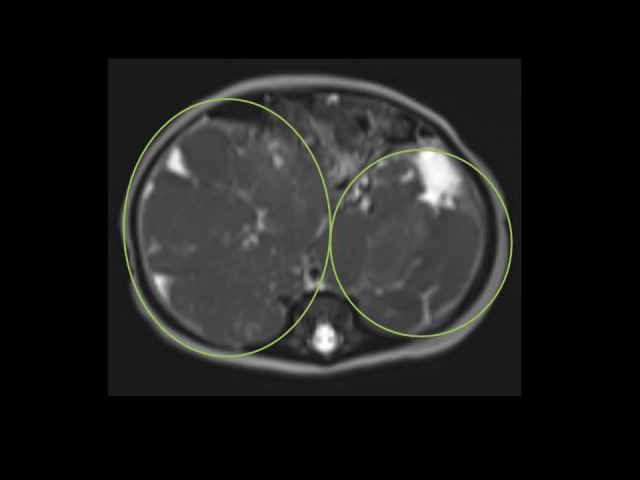
Bilateral Ewing sarcoma/primitive neuroectodermal tumor: axial T2 HASTE image through upper abdomen demonstrates hypointense bilateral lobulated giant masses arising and infiltrating both kidneys (circles).

Over a 23-year period, 20 children with NWRTs were identified. The major histological groups were malignant rhabdoid tumor (Figure 1) (25%), mesoblastic nephroma (Figure 2) (25%), renal cell carcinoma (15%), inflammatory myofibroblastic tumor (10%), multilocular cystic renal tumors (10%), metanephric adenoma (5%), renal neuroblastoma (Figure 3) (5%), and bilateral ES/PNET (Figure 4). The patients’ demographics and clinical signs are summarized in the Table. There was a male predominance (female/male: 7/13). Female patients had mesoblastic nephroma, renal cell carcinoma, renal neuroblastoma, and bilateral renal ES/PNET. Median age at diagnosis was 3.2 years old (0.1–13.5 years old). The most commonly seen presenting symptoms were abdominal mass, flank pain, and hematuria. None of the patients had hypertension. In imaging studies there were no renal vena thromboses. Median tumor diameter was 8.9 cm (3–15 cm). Among the 20 children, only two patients with malignant rhabdoid tumor and renal cell carcinoma had distant metastasis at the time of diagnosis. One of the children with an inflammatory myofibroblastic tumor with both renal and pulmonary involvement was reported previously [9]. All of the patients with NWRTs had radical nephrectomy except the child with bilateral renal ES/PNET. Adjuvant chemotherapy was given to patients with malignant rhabdoid tumor, mesoblastic nephroma, and bilateral renal ES/PNET. Different chemotherapy protocols were used for each type of tumors. Children with mesoblastic nephroma were treated similar to cases of Wilms tumor, receiving actinomycin-D (15 μg/kg, intravenous (iv), on days 1–5), vincristine (1.5 mg/m2 iv on day 1), and doxorubicin (50 mg/m2 iv over 4 h on day 1). Those with malignant rhabdoid tumors were managed with ifosfamide (2000 mg/m2 iv on days 2, 3, and 4), carboplatin (targeted to an area under the curve of 6 mg/mL per minute, iv, on day 1), and etoposide (100 mg/m2 iv on days 2, 3, and 4), alternating vincristine (1.5 mg/m2 iv on days 1 and 8), doxorubicin (75 mg/m2 iv over 48 h from day 1), and cyclophosphamide (1500 mg/m2 iv on day 1). In addition, one child with ES/PNET received doxorubicin (20 mg/m2 iv, days 1–3) and etoposide (100 mg/m2 iv, days 1–3). Two of the patients with mesoblastic nephroma received abdominal radiotherapy. One of the patient with inflammatory myofibroblastic tumor was treated with crizotinib. Two other patients with mesoblastic nephroma, three of the patients with rhabdoid tumor, and the child with bilateral renal ES/PNET died because of progression. The mortality rate was 30%.

**Table 1:** Patients demographics and clinical signs.

Patient no.	Sex	Age(years)	Presentation	Histology	Stage	Tumor diameter (cm)	Outcomes
1	M	3.5	Anorexia, abdominal mass, anemia	Mesoblastic nephroma	I	9	Exitus
2	F	0.5	Abdominal mass		I	10	Alive
3	M	2	Flank pain, abdominal mass		I	10	Exitus
4	F	0.1	Abdominal mass		I	10	Alive
5	F	1	Abdominal mass		I	10	Alive
6	M	3	Abdominal mass	Malignant rhabdoid tumor	IV	7.5	Exitus
7	M	0.5	Hematuria, abdominal mass		I	9	Exitus
8910	MMM	232	HematuriaHematuria Hematuria		IIVI	3153	AliveExitusAlive
11	F	13.5	Flank pain, abdominal mass	Renal cell carcinoma	I	10	Alive
12	M	6	Hematuria, abdominal mass		I	13	Alive
13	M	11	Flank pain, weight loss		IV	8	Alive
14	M	3	Anorexia	Inflammatory myofibroblastic tumor	IV	6	Alive
15	M	0.5	Hematuria, anemia		I	9.5	Alive
16	M	1.5	Abdominal mass	Multilocular cystic renal tumor	I	10	Alive
17	M	1.5	Abdominal mass		I	9	Alive
18	M	7.5	Anorexia	Metanephric adenoma	I	4	Alive
19	F	2	Abdominal mass	Renal neuroblastoma	I	6	Alive
20	F	0.2	Abdominal mass	Bilateral Ewing sarcoma/PNET	V	Left: 15, right: 15	Alive

## 4. Discussion 

Pediatric NWRTs constitute a very small part of childhood malignancies. Although they are very rare tumors, it is important to define the diagnosis and start adequate treatment immediately because of the high morbidity and mortality rates. In this group, local stage and radical resection are generally associated with increased survival. Mesoblastic nephroma, malignant rhabdoid tumor, renal cell carcinoma, inflammatory myofibroblastic tumor, multilocular cystic renal tumors, and metanephric adenoma are the most common tumors among pediatric NWRTs. Renal neuroblastoma and bilateral renal ES/PNET are extremely rare entities among NWRTs [3,4,10]. 

Mesoblastic nephroma is the most frequent type of NWRT in the neonatal period and 90% percent of patients are seen under the age of 3 months old. Total resection of the tumor is usually curative, but local recurrences can be seen [11–13]. Cellular variants and high mitotic indexes of mesoblastic nephroma have poor outcomes with bone and brain metastases and local recurrence [12,13]. Patients older than 3 months old, those with cellular variants, and those with residual tumors may particularly benefit from chemotherapy. Two patients in our cohort with mesoblastic nephroma died because of relapse after the end of the chemotherapy. This may have been related to the patients’ ages (2.5 and 3.5 years old) and tumor sizes (maximal diameters were >10 cm). 

Malignant rhabdoid tumor of the kidney is a rare aggressive cancer, occurring in infancy and early childhood and accounting for only 2% of all renal tumors in childhood [14]. Patients with malignant rhabdoid tumor of the kidney are characterized by young age and advanced stage at presentation. Metastases are usually to the lungs and brain. Hematuria, fever, infection, anemia, and hypertension are the most common presenting symptoms. Prognostic factors are sex, age at diagnosis, tumor stage, and presence or absence of CNS lesions [15]. Patients under the age of 2 years have the worst prognosis because in this group relapses occur early and patients have a tendency to develop CNS tumors at the same time [14,15]. Hematuria is the most common presenting symptom. Four of five of our patients had hematuria at the time of diagnosis. Chemotherapy and radiation therapy sensitivity is low but carboplatin, etoposide, doxorubicin, and vincristine have been reported for multiagent chemotherapy. One of our patients, who was 3 years old with a tumor of 7.5 cm in diameter and stage 4 disease, died during chemotherapy. A 6-month-old male patient with a mass of 9 cm in diameter died 2 months after the completion of chemotherapy. A 3-year-old boy with a tumor of 15 cm in diameter and stage 4 disease died while receiving chemotherapy because of progressive disease with pulmonary metastasis and septic shock.

Renal cell carcinoma (RCC) is a rare pediatric tumor that accounts for approximately 2% of all pediatric renal tumors, while it is the most common renal tumor in adults [16–19]. Children with RCC generally present at 9–15 years of age; the ages of the presented children in the current study at diagnosis were 5, 6, 11, and 13 years. Our patients mostly presented with symptoms of hematuria, flank pain, and abdominal mass. As in other subtypes of NWRTs, patients with localized tumors have a good prognosis, whereas prognosis in metastatic cases is poor. Renal medullary carcinomas are highly aggressive tumors and commonly present as enlarged cervical lymph nodes. Surgical resection of the tumor with radical nephrectomy is the mainstay of treatment. Adjuvant therapy is not recommended for children with microphthalmia transcription factor translocation RCC, papillary RCC, or no residual tumor after resection [16–19]. No effective therapy for disseminated disease is available. We did not give adjuvant chemotherapy to any children with RCC because of complete tumor resection after surgery. 

Inflammatory myofibroblastic tumor is a very rare benign reactive proliferative lesion. It is rarely seen in the urinary tract. It has a low recurrence rate and rarely metastasizes [9,20,21]. The lung is the most common site of the tumor. In the urinary tract, inflammatory myofibroblastic tumors most commonly occur in the urinary bladder [20]. Diagnosis should be confirmed by histopathological study [21]. The fundamental treatment is complete surgical resection. Local recurrence, malignant transformation, and metastasis have been reported rarely. Metastatic or recurrent cases should be treated with corticosteroids. Approximately half of inflammatory myofibroblastic tumors carry rearrangements of the anaplastic lymphoma kinase. The inhibitor of anaplastic lymphoma kinase inhibitor, crizotinib, has been used for these patients [4]. One of the reported patients with inflammatory myofibroblastic tumor had received crizotinib and showed a complete response [9].

Multicystic nephroma and cystic partially differentiated nephroblastoma are two histological subtypes [22–24]. These are nonheritable, benign lesions and are rare both in children and adults [23]. Most children present with a painless abdominal mass. The sonographic appearance of the multilocular cystic renal tumor includes multiple anechoic spaces traversed by thin septa and no solid elements [22–24]. No cases of aggressive behavior have been documented, but surgery is required for the diagnosis and differential diagnosis from cystic Wilms tumor, RCC, or mesoblastic nephroma [4]. Recurrence can occurred following incomplete resection. Two of our children with NWRTs were diagnosed with multicystic nephroma and showed remission after complete resection.

Metanephric adenoma is a rare benign neoplasm, uncommonly seen in the pediatric population. These are usually asymptomatic lesions detected incidentally in imaging studies performed for other indications [25,26]. They generally appear as hypovascularized solid lesions. Diagnosis is made histologically. Partial nephrectomy is curative. Metastatic disease is very rare but should be considered [25]. Only one child was diagnosed with metanephric adenoma in this cohort and showed remission after complete nephrectomy.

Primary intrarenal neuroblastoma is a rare condition. Intrarenal neuroblastoma typically results from direct renal invasion from an adrenal neuroblastoma, but true intrarenal neuroblastoma originates from either sequestered adrenal rests during fetal life or intrarenal sympathetic ganglia [27,28]. From our cohort of children with NWRTs, only a 2-year-old girl was diagnosed with primary intrarenal neuroblastoma. She was treated according to the national neuroblastoma protocol entitled “Turkish Pediatric Oncology Group Neuroblastoma Protocol 2009” as described by Ozguven et al. [29]. Our case was not MYCN-amplified and showed remission after complete nephrectomy without further chemotherapy.

ES/PNET is a high-grade malignant neoplasm commonly affecting the bones of the thoracic region. Primary ES/PNET of the kidney is extremely rare; it commonly affects young adults and is rarely reported in young children [10,30]. Our patient was a 3-month-old female with bilateral renal involvement. The pathological morphology was relevant for renal ES/PNET and FISH for *EWSR1* was positive. To the best of our knowledge, bilateral renal involvement of ES/PNET was not previously reported in the English medical literature. 

Ultrasound-guided renal biopsies showed high effectiveness and safety but we did not perform any renal biopsies in the presented children with NWRTs to avoid upstaging of the disease [31]. Surgical nephrectomies were performed after the discussion of each individual case with multidisciplinary pediatric oncology councils. 

In conclusion, our series of pediatric NWRTs consists of 18.7% of all pediatric renal tumors. In this rare group of tumors, the mortality rate was found to be 30% in our series. Clinicians must be vigilant about the different properties of this heterogenic group of tumors. 

## Acknowledgments

The authors thank Fatma Türkan Mutlu, Özlem Gül Kırkaş, Şefika Akyol (Pediatric Oncology), and S. Burcu Görkem (Pediatric Radiology) for their contributions to the reported cases.
